# Inhibition of Dorsal Root Ganglia Transient Receptor Potential Ankyrin 1 Upregulation Contributes to the Protective Effect of Morphine Against Gastric Mucosal Damage Induced by Water-Immersion Restraint Stress

**DOI:** 10.5152/tjg.2024.23267

**Published:** 2024-06-01

**Authors:** Qun Jiang, Peng Jiang, Mingyan Guo, Chuangbo Xie, Qiong Ling, Gaofeng Zhao, Weifeng Tu, Xiangyu Li

**Affiliations:** 1 The First School of Clinical Medicine, Southern Medical University, Guangzhou, China; 2 Department of Anesthesiology, The Second Affiliated Hospital of Guangzhou University of Chinese Medicine, Guangzhou, China; 3 Department of Anesthesiology, Huizhou Municipal Central Hospital, Huizhou, China; 4 Department of Anesthesiology, Sun Yat-sen Memorial Hospital, Sun Yat-sen University, Guangzhou, China; 5 The Center of Anesthesiology and Perioperative Medicine, Jinshazhou Hospital of Guangzhou University of Chinese Medicine, Guangzhou, China

**Keywords:** Acute gastric mucosal lesion, morphine, substance P, TRPA1 channel, water-immersion restraint stress

**Main Points**

- We demonstrated that water-immersion restraint stress (WIRS) upregulated transient receptor potential ankyrin 1 (TRPA1) and substance P (SP) levels in the gastric mucosa. The administration of a TRPA1 antagonist (HC-030031) or a protein kinase A inhibitor alleviated gastric mucosal lesions caused by WIRS, suggesting the involvement of TRPA1 signaling in acute gastric mucosal lesions (AGML).
- Intrathecal morphine administration was found to suppress WIRS-induced gastric mucosal lesions and the upregulation of TRPA1/SP, emphasizing the protective potential of morphine preconditioning.
- Our study revealed that TRPA1 and SP play crucial roles in the pathogenesis of WIRS-induced AGML and that exogenous gastroprotective strategies can modulate TRPA1 levels via the cyclic adenosine monophosphate/protein kinase A-dependent pathway.
- Inhibition of TRPA1 upregulation in the dorsal root ganglia emerged as a critical mechanism underlying the protective effects of intrathecal morphine preconditioning.

## Introduction

Acute gastric mucosal lesion (AGML) is a stress-induced gastric mucosal disease caused by prolonged anxiety, stress, sepsis, surgery, and various trauma.^1^ Patients with stress-induced gastric ulcers tend to be at a higher risk of ulcer bleeding,^2,3^ which in turn leads to an increased risk of mortality.^4,5^ As the pathogenesis mechanism of AGML is still unclear,^6^ further exploration is urgently needed to find a new therapeutic target.

Morphine is a specific µ-opioid receptor (MOR) agonist that we found to play a crucial protective role in water-immersion restraint stress (WIRS)-induced AGML based on our previous study. The interaction between MOR and the transient receptor potential vanilloid 1 (TRPV1) receptor is particularly noteworthy in the context of antinociception.^7,8^ Both TRPV1 and transient receptor potential ankyrin 1 (TRPA1) receptors are essential for inflammation-induced hyperalgesia, which is an increased sensitivity to painful stimuli that occurs as a result of tissue inflammation.^9^ The TRPA1 is a noxious cold sensor that helps to transmit the noxious cold signal to the central neurons in the spinal cord.^10^ Both TRPV1 and TRPA1 are highly co-expressed in different regions of the central nervous system.^11,12^ These specialized sensory neurons serve crucial functions in sensing temperature, pressure, and chemicals, as well as contributing to neurogenic inflammation and hyperalgesia,^13-15^ and it is suggested that these receptors may interact in a cyclic adenosine monophosphate/protein kinase A system (cAMP/PKA)-dependent manner.^16,17^ Thus, the current study assumed that there might be functional interactions between TRPA1 and MOR. Both TRPV1 and TRPA1 contribute to gastric function.^18-21^ Unlike TRPV1, TRPA1 is less studied, and little is known about its role in the stomach.^22^

Our previous results showed opioids that activate the µ-opioid receptor attenuated WIRS-induced gastric mucosal lesions in rats.^23^ Multiple studies have demonstrated the association between opioid receptors and TRPV1.^24,25^ According to the results of our previous research, TRPA1 has a significant role in the progression of AGML induced by WIRS.^26^

The manifestation of TRPV1 and TRPA1 has been studied in stomach-reflected thoracic dorsal root ganglia (DRG).^27,28^ The current study speculated that there may be a functional interaction between TRPA1 and MOR. The objective of this study is to delve deeper into the protective influence and the underlying mechanism of intrathecal morphine preconditioning (ITMP) on AGML induced by stress. This study aimed to investigate whether morphine might regulate the expression and function of TRPA1 through a cAMP/PKA-dependent pathway, thereby alleviating gastric mucosal lesions caused by WIRS.

## Materials and Methods

### Animals

During the experiments, male Wistar rats aged 8-12 weeks with a weight between 180 and 220 g were chosen. These rats were sourced from the Experimental Animal Center of South Medical University. The animals were kept under controlled conditions with appropriate temperature and humidity, and they were subjected to a light/dark cycle of 12 hours each. Prior to the experiments, the rats were subjected to a 24-hour fasting period with free access to water. All experimental protocols involving the use of animals were conducted following the guidelines and regulations for the ethical utilization of laboratory animals. The study received approval from the Ethics Committee for the Use of Experimental Animals at the Second Affiliated Hospital of Guangzhou University of Traditional Chinese Medicine (Approval no: 2020029, Date: 29/12/2020).

### Implantation of the Intrathecal Catheter

The intrathecal tube implant model was set up based on a method described in previous studies.^29,30^ In summary, rats were anesthetized using an intraperitoneal injection of 60 mg/kg sodium pentobarbitone (Sigma, USA). The skin covering the L2 to S1 vertebrae was trimmed and then disinfected using povidone iodine. A small hole measuring 2×2 mm was made in the atlantooccipital membrane. With the assistance of a 12-gauge needle, a polyethylene 10 catheter measuring 4 cm in length was subsequently introduced into the thoracic spinal cord. The incision was then sutured using interrupted stitches on the skin. After the procedure, rats were placed back into individual enclosures in the animal room to facilitate their recovery after the operation. Rats displaying dyskinesia were not included in subsequent experiments. The remaining animals received an intrathecal administration of 2% Lidocaine (10 µL) to verify the effectiveness of the injection. Successful injection was confirmed by observing hind leg dyskinesia as a sign of successful administration.

### Animal Group and Water-Immersion Restraint Stress Model

The rats with intrathecal tubes were divided into 6 groups, including the control group, WIRS group, WIRS + ITMP group, WIRS + HC-030031 group, WIRS + ITMP + µ-opioid receptor antagonist (CTAP) group, and WIRS+H89 group. The rats in the control group did not have an operation performed on them. In the WIRS group, the rats were restrained in special devices and then submerged in a water bath at 20 ± 1℃, up to the xiphoid for 6 hours to cause AGML formation.^26,31^ Prior to WIRS, ITMP was administered with 3 consecutive 5-minute intrathecal infusions of morphine (3 µg/kg, 10 µL), separated by 5-minute periods without infusion. HC-030031 (a specific TRPA1 antagonist, 1ug/µL, 10µL), CTAP (a specific µ-opioid receptor antagonist, 1 µg/µL, 10 µL), and H89 (a specific PKA inhibitor, 4 nM) were also intrathecally administered 30 minutes prior to the induction of WIRS.

### Tissue Harvest and Lesion Index Calculation

Following the experiment, all of the animal stomachs were dissected and opened along the greater curvature. These stomachs were subsequently washed with saline and subjected to thorough examination for any mucosal lesions. To maintain objectivity, the quantification of lesions was performed in a blind manner using the lesion index calculation according to Guth et al’s^32^ standard.

### Hematoxylin and Eosin Staining

The stomachs and thoracic DRGs from segments T8 to T12 were carefully dissected and then fixed by 4% paraformaldehyde overnight. Afterward, the tissues were embedded in paraffin for further processing. Gastric tissue sections (3 µm) were obtained and stained with histological alterations in gastric lesions; for this, a semi-quantitative scoring system known as the histopathological gastric lesion score (HGS) was employed.^33^ The HGS evaluated various factors such as ulceration, mucosal damage, glandular atrophy, and reduction. Hyperemia/edema, as well as inflammatory cell infiltration and submucosal edema, were assessed using a scoring system with a maximum of 10 points (Table 1). Based on a modified version of a known system, HGS serves as a valuable tool for evaluating experimental AGML.

### Histological Studies

Gastric tissue measuring about 1 mm^3^ was fixed in 2.5% phosphate-buffered glutaraldehyde (pH 7.2, 0.1M) immediately after being isolated overnight at a temperature of 4℃. Post fixation was conducted using 1% phosphate-buffered osmium tetroxide for 1 hour. Following fixation, gradual dehydration was performed by increasing concentrations of alcohol. The tissue samples that had undergone dehydration were subsequently embedded into epoxy resin. Thin sections were meticulously generated from each specimen and then subjected to staining with uranyl acetate and citrate to improve contrast for better visualization under a transmission electron microscope. For each experimental group, 2 randomly selected grids were examined using the transmission electron microscope. To assess cellular damage, 5 specific criteria were utilized: (i) whether perinuclear space with nuclear damage was present (1) or absent (0); (ii) whether there was mitochondrial damage (1) or not (0); (iii) if there was dilatation in the granular endoplasmic reticulum (GER) (1) or not (0); (iv) if vacuoles were formed in the GER (1) or not (0); (v) whether tight junctions were loosened (1) or not (0). Each of these criteria was evaluated using a binary rating of 1 or 0, allowing for a maximum possible total rating of 5 for each individual slide.

### Antibodies and Enzyme-Linked Immunosorbent Assay Kits

For immunofluorescence and immunohistochemistry, rabbit anti-TRPA1 #DF13269 (1:200, Affinity, Ohio, USA), rabbit anti-μ-receptor #bs-3623R (1:400, Bioss, Beijing, China), rabbit anti-substance P #bs-0065R (1:400, Bioss, Beijing, China), goat anti-rabbit conjugated with Fluorescein Isothiocyanate (FITC) #BA1105 (1:100, Boster, Wuhan, China) and goat anti-rabbit conjugated with Cyanine (Cy3) #BA1032 (1:100, Boster, Wuhan, China) were used. For Western blot, TRPA1 #DF13269 (1:500, Affinity, Ohio,USA) and Glyceraldehyde3-phosphate dehydrogenase (GAPDH) #BM1623 (1000, Boster, Wuhan, China) were used. For enzyme-linked immunosorbent assay (ELISA), ELISA kits for SP #KGE007 (R&D Systems, Minnesota, USA) and CAMP #CEC419Ra (Cloud-Clone Corp, Texas, USA) were used.

### Immunofluorescence

Thoracic DRG (T8-T12) was collected, fixed in 4% paraformaldehyde solution overnight at 4°C. Afterward, the tissues were embedded in Tissue-Tek compound (Bayer Healthcare, Pittsburgh, Pa, USA), and consecutive 10 μm sections were prepared using a cryostat and mounted onto slides coated with gelatin to minimize nonspecific binding. The slices were subjected to incubation at 4°C overnight with a rabbit anti-TRPA1 antibody and rabbit anti-μ-receptor, followed by goat anti-rabbit conjugated with FITC and goat anti-rabbit conjugated with Cy3 for 40 minutes at 37℃ to label the respective targets. Following the incubation steps, the sections underwent PBS rinsing, followed by mounting in a mounting medium, and subjected to examination using a confocal laser scanning microscope (Leica, Germany). The imaging process was conducted by an experimenter who was unaware of the treatment protocol, ensuring unbiased data analysis. The excitation settings for the confocal laser scanning microscope were kept consistent during the analysis.

### Quantitative Real-Time Polymerase Chain Reaction

Extraction of total RNA from T8-T12 DRG tissue was done on both sides of the model rats. Tissue samples were also obtained from untreated rats to establish a baseline for comparison. TRIzol (Invitrogen, USA) was used to extract RNA from the DRG. Real-time polymerase chain reaction (PCR) was conducted following the manufacturer’s instructions for the specific biological system. For mRNA analysis, a total of 400 ng RNA was converted into cDNA through reverse transcription, followed by amplification using the SYBR Green method. The TRPA1 gene was amplified using the following primers: forward 5′-AACAATGCTCTGGAGTGGGT-3′ and reverse 5′-CTCCCGTCGATCTCAGCAAT-3′. As a reference gene, beta-actin was utilized, and the following primers were employed: forward 5′- GATCAAGATCATTGCTCCTCCTG-3′ and reverse 5′-AGGGTGTAAAACGCAGCTCA-3′. The amplification process comprised 40 cycles. The β-actin gene transcript served as an internal control for gene expression measurements, employing the comparative CT (ΔΔCT) method. The PCR products, along with a size marker DNA Marker XIV (Takara, Japan), were separated through electrophoresis on a 2% agarose gel infused with ethidium bromide (Sigma, Taufkirchen, Germany) and their visualization was achieved under UV light (Bio-Rad, USA). The PCR products were subjected to sequencing bidirectionally (AGOWA, Berlin, Germany), and the resulting sequences were then compared with the established published sequences of TRPA1.

### Western Blot Analysis

Dorsal root ganglia tissues (T8-T12 segments) were collected simultaneously with the same time periods as those employed for immunohistochemistry investigations. The protein concentration in the homogenate was assessed using a microplate reader and a bicinchoninic acid kit. To ensure the accuracy of the analysis, equivalent amounts of protein (20 μg) were separated using 7.5% sodium dodecyl sulfate–polyacrylamide gels (Fisher Scientific) and subsequently transferred to a polyvinylidene difluoride (PVDF) membrane (Invitrogen). The PVDF membranes were subjected to blocking with 5% non-fat dry milk for a duration of 1 hour in Tris-buffered saline that contained Tween 20. Subsequently, the membranes were subjected to an overnight incubation at a temperature of 4°C with primary antibodies specifically against TRPA1 and GAPDH. The PVDF membranes were subsequently exposed to a 2-hour incubation with a horseradish peroxidase-conjugated goat anti-mouse antibody. Normalization of the protein for each sample was performed by comparing it to the level of GAPDH.

### Immunohistochemistry

For immunohistochemistry (IHC) analysis, sections of gastric tissue with a thickness of 3 μm underwent a dewaxing process using Bio-Clear, followed by hydration through an ethanol gradient. Subsequently, the sections were subjected to incubation with antibodies, including the rabbit anti-TRPA1 and rabbit Anti-Substance P antibody overnight at 4℃. A secondary antibody, coupled with the poly-HRP rabbit detection system (E-IR-R217), was used for visualization. Sections were examined and visualized using a BX51 fluorescent digital imaging microscope from Olympus, Japan. To quantitate the IHC signals in the DRG, the optical density values were assessed using Image-Pro Plus 6.0 software.

### Enzyme-Linked Immunosorbent Assay

Dorsal root ganglia tissue samples were collected from T8 to T12 segments and then homogenized in 3 mL of 2 N acetic acid. After homogenization, the samples were centrifuged at 4°C, 4500 *g* for 10 minutes. The collected supernatant was used for the evaluation of SP and cAMP levels through specialized ELISA kits. The quantification of sample contents was performed in accordance with the guidelines provided by the manufacturer. The results are displayed as micrograms per gram of protein.

### Statistical Analysis

All data were expressed as means ± SEM. Statistical analysis was conducted using one-way analysis of varriance, followed by a Student’s *t*-test to assess the group difference. A significance threshold of *P *< .05 was employed for all statistical tests.

## Results

### Transient Receptor Potential Ankyrin 1 Antagonist HC-030031 and Protein Kinase A Inhibitor H-89 Mitigated Water-Immersion Restraint Stress-Induced Gastric Lesions

**Figure 1.**
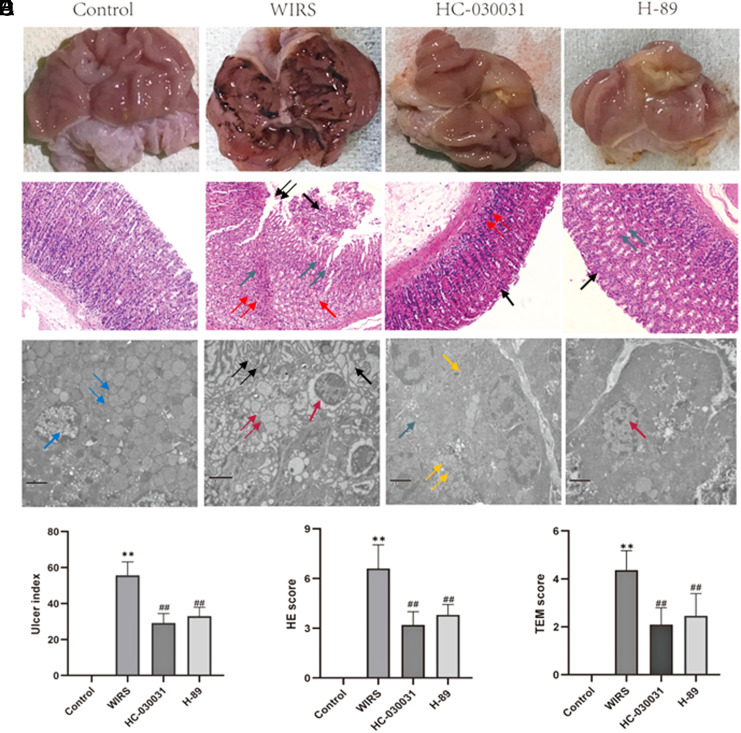
Gastric mucosal lesions induced by WIRS and the protective effects of HC-030031 and H-89. (A) Representative gross macroscopic appearance of the gastric mucosa. (B) Histopathology of gastric sections stained by H&E. The histopathological features were shown as follows: single black arrow indicates hemorrhagic necrosis; double green arrows show edema; single red arrow indicates gland atrophy and reduction; double red arrows show inflammatory cell infiltration. (C) Histopathology images of stomach sections taken by a transmission electron microscope. The histopathological features were shown as follows: single blue arrow indicates nucleus, double blue arrows show mitochondria; single red arrow indicate perinuclear space with nuclear damage; double red arrows show loss of mitochondrial cristae and disapperance of mitochondrial structure; single black arrow indicates tight juction loosing; double black arrows show vacuoles in GER; single yellow arrow indicates GER; double yellow arrows show partial.(D) Gastric lesions were observed, and the ulcer indices are shown. (E) H&E scores are shown. (F) Transmission Electron Microscopy (TEM) scores are shown. All data are presented as mean ± SEM, n = 6 rats per group; statistical analysis was performed using a Student’s *t*-test, **P < .0001 compared to the control group; ##*P *< .01 compared to the WIRS group. H&E, hematoxylin and eosin; WIRS, water-immersion restraint stress.

After 6 hours of WIRS, gastric tissues were excised, and the gastric lesions index was observed and calculated. In the control group, the rats had smooth and intact mucosa, while in all other groups, WIRS caused noticeable hemorrhaging on the luminal surface of the stomach, along with a significant area of diffuse reddening observed throughout the mucosal lining; the calculation of ulcer index was increased from 0 to 52.7 ± 8.19 (*P* < .0001, Figure 1A and D).

Histological analysis demonstrated that WIRS resulted in substantial damage to the stomachs of the rats. The observed damage encompassed acute erosive bleeding lesions characterized by extensive coagulative cell necrosis, pronounced vascular congestion, multiple superficial erosions, extravasation of erythrocytes, necrosis, submucosal edema, as well as scattered inflammatory cells, primarily neutrophils, that were evident within the deep mucosal and submucosal layers. Water-immersion restraint stress led to the emergence of multiple gastric mucosal lesions, and the calculation of the H&E score was increased from 0 to 6.9 ± 1.37 (*P* < .0001, Figure 1B and 1E).

On a transmission electron microscope, the WIRS groups displayed several distinctive features. These manifestations encompassed the existence of vacuoles within the GER cisterns, loosening and disruption of perinuclear tight junctions, damage to gastric epithelial cells, excessive secretion resulting in tubule dilation, the absence of mitochondrial cristae, and evident damage to mitochondria in parietal cells. The calculation of the TEM score increased from 0 to 4.36 ± 0.8 (*P* < .0001, Figure 1C and 1F).

To explore the role of TRPA1 in WIRS, HC-030031 was administered intrathecally to inhibit TRPA1 in the DRG. Following the administration of HC-030031, the ulcer index of gastric mucosa was significantly reduced from 52.7 ± 8.19 to 25.2 ± 6.28 (*P* < .0001, Figure 1A and D). The HGS decreased from 6.9 ± 1.37 to 3.6 ± 1.07 (*P* < .0001, Figure 1B and 1E), and the GER decreased from 4.36 ± 0.8 to 2.09 ± 0.7 (*P* < .0001, Figure 1C and 1F).

The cAMP signaling pathway, it was found that WIRS led to an increase in the level of cAMP in the DRG from 120.7 ± 13.51 to 232.7 ± 42.7 (*P* = .0024, Figure 5F). Intrathecal administration of H-89 was observed to reduce gastric mucosal lesions induced by WIRS. The ulcer index decreased from 52.7 ± 8.19 to 32.9 ± 5.02 (*P* < .0001, Figure 1D). The HGS was reduced from 6.9 ± 1.37 to 3.8 ± 0.63 (*P* < .0001, Figure 1E), and the GER decreased from 4.36 ± 0.8 to 2.45 ± 0.93 (*P* < .0001, Figure 1F).

### Water-Immersion Restraint Stress Induced Upregulation of Transient Receptor Potential Ankyrin 1 and Substance P in the Dorsal Root Ganglia

**Figure 2.**
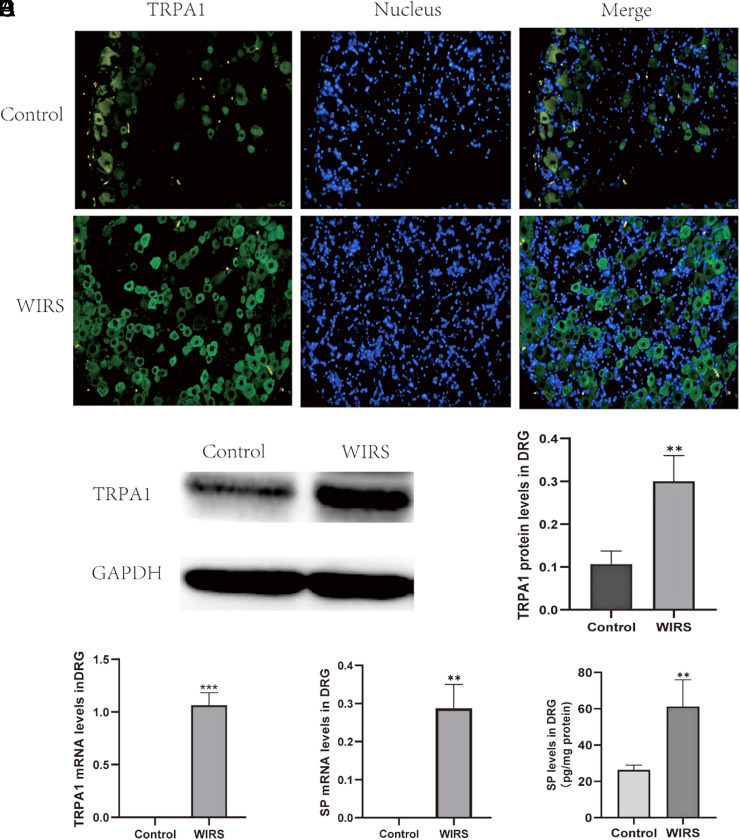
The upregulated expression of TRPA1 and SP in the DRG induced by WIRS. (A) The distribution of TRPA1 in the thoracic gastric reflected the DRG after WIRS was demonstrated by immunofluorescence. The green fluorescence represented TRPA1, while the blue fluorescence represented the nuclei. (B, C) Relative TRPA1 protein levels in the DRG as measured by Western blot. (D) TRPA1 mRNA levels in the DRG as measured by real-time-PCR. (E) SP mRNA levels in the DRG as measured by real-time-PCR. (F) SP levels in the DRG as measured by ELISA. All data are presented as mean ± SEM, n = 3 rats per group; statistical analysis was performed using a student’s *t*-test, ***P < .001, **P < .01; compared to the control group. DRG, dorsal root ganglia; PCR, polymerase chain reaction; SP, substance P; TRPA1, transient receptor potential ankyrin 1; WIRS, water-immersion restraint stress.

Real-time PCR was employed to confirm the specificity of the primers for TRPA1 and SP. Relative quantification of TRPA1 mRNA and SP mRNA showed that they were significantly increased after WIRS. The data are presented as the percentage change relative to the control group (Figure 2D and 2E). Western blot results showed the relative density of TRPV1 protein immunoblots from T8-T12 DRG tissue after WIRS (Figure 2B). The TRPA1 relative density exhibited a significant increase after WIRS in comparison to the control group from 0.11 ± 0.03 to 0.30 ± 0.06 (*P* = .0076, Figure 2C). The upregulation of SP in the DRG was confirmed by ELISA; the protein levels of SP in the DRG increased from 26.32 ± 2.63 to 61.16 ± 14.75 (*P* = .0035, Figure 2F). In addition, immunofluorescence was used to assess the TRPA1 expression in the DRG. Consistent with prior findings, an increase in the fluorescence intensity of TRPA1 was observed to be heightened after WIRS (Figure 2A).

### Water-Immersion Restraint Stress Induced Upregulation of Transient Receptor Potential Ankyrin 1 and Substance P in the gastric mucosa

**Figure 3.**
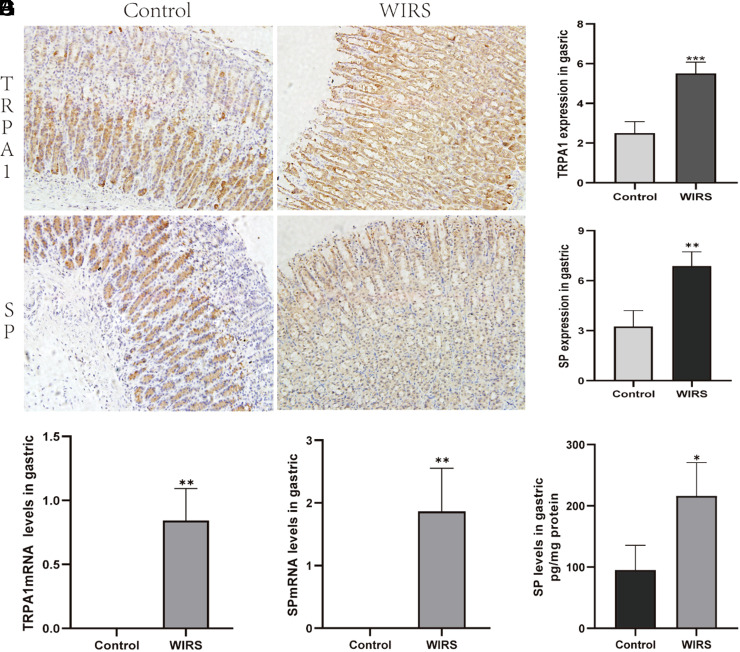
The upregulated expression of TRPA1 and SP in the gastric mucosa induced by WIRS. (A) Distribution of TRPA1 in the gastric mucosa as demonstrated by IHC. (B) Distribution of SP in the gastric mucosa as demonstrated by IHC. (C) The upregulated expression of TRPA1 as measured by IHC. (D) The upregulated expression of SP as measured by IHC. (E) TRPA1 mRNA levels in the gastric mucosa as measured by real-time-PCR. (F) SP mRNA levels in the gastric mucosa as measured by real-timee-PCR. (G) SP levels in the gastric mucosa as measured by ELISA. All data are presented as mean ± SEM, n = 3 rats per group; statistical analysis was performed using a student’s t-test, ***P < .001, **P < .01, *P < .05 compared to the control group. IHC, immunohistochemistry; PCR, polymerase chain reaction; SP, substance P; TRPA1, transient receptor potential ankyrin 1; WIRS, water-immersion restraint stress.

According to IHC results, an elevation in TRPA1 expression in the gastric mucosa was observed in the WIRS group compared to the control group (*P* = .0003, Figure 3A and 3C). Real-time PCR further confirmed that TRPA1 mRNA levels were significantly enhanced by WIRS (Figure 3E). Water-immersion restraint stress induced a significant release of SP in the stomach (*P* = .0013, Figure 3B and 3D), and this was further corroborated by real-time PCR. The data showed that SP mRNA levels markedly increased after WIRS in comparison with the control group (Figure 3F). A similar tendency was observed in the ELISA result, where the SP protein levels in the gastric mucosa increased from 95.07 ± 40.52 to 216 ± 54.55 (*P* = .012, Figure 3G).

### Suppression of Water-Immersion Restraint Stress-Induced Gastric Mucosal Lesions and Transient Receptor Potential Ankyrin 1/Substance P Upregulation by Morphine

**Figure 4.**
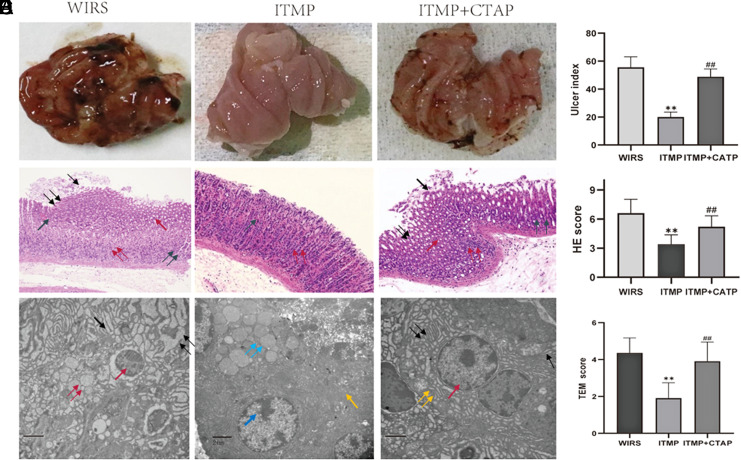
ITMP pretreatment attenuated WIRS-induced gastric injury, but the protective effect could be reduced when combined with CTAP. (A) Representative gross macroscopic appearance of the gastric mucosa. (B) Histopathology of gastric sections stained by H&E. The histopathological features were shown as Figure1B. (C) Histopathology images of stomach sections taken by a transmission electron microscope. (D) Gastric lesions were observed, and the ulcer indices are shown. (E) H&E scores are shown. (F) TEM scores are shown. All data are presented as mean ± SEM, n = 6 rats per group; statistical analysis was performed using a student’s *t*-test, **P < .01 compared to the WIRS group; ##*P*< .01 compared to the ITMP group. CTAP, µ-opioid receptor antagonist; ITMP, intrathecal morphine preconditioning; WIRS, water-immersion restraint stress.

We found that ITMP had a consistent protective effect on gastric mucosa against WIRS by reducing the ulcer index from 52.7 ± 8.19 to 20.7 ± 3.4 (*P*< .0001, Figure 4A and 4D) and the HGS from 6.9 ± 1.37 to 3.4 ± 0.9 (*P* < .0001, Figure 4B and 4E) respectively. This protective effect was also confirmed by transmission electron microscopy since the TEM score was reduced from 4.36 ± 0.8 to 1.9 ± 0.83 (*P* < .0001, Figure 4C and 4F). Administration of CTAP intrathecally at a dose of 1µg/µL, 10µL 10 minutes before ITMP, resulted in a reduction of the effect of ITMP on the gastric mucosa. The ulcer index increased from 20.7 ± 3.48 to 48.8 ± 5.5 (*P* < .0001, Figure 4A and 4D) and the HGS from 3.4 ± 0.9 to 5.2 ± 1.14 (*P* < .0001, Figure 4B and 4E). This inhibiting effect was also confirmed by transmission electron microscopy; the TEM score increased from 1.9 ± 0.83 to 3.9 ± 1.04 (*P* < .0001, Figure 4C and 4F). The statistical analysis also revealed that there was no noteworthy difference between the CTAP + ITMP group and the WIRS group.

**Figure 5.**
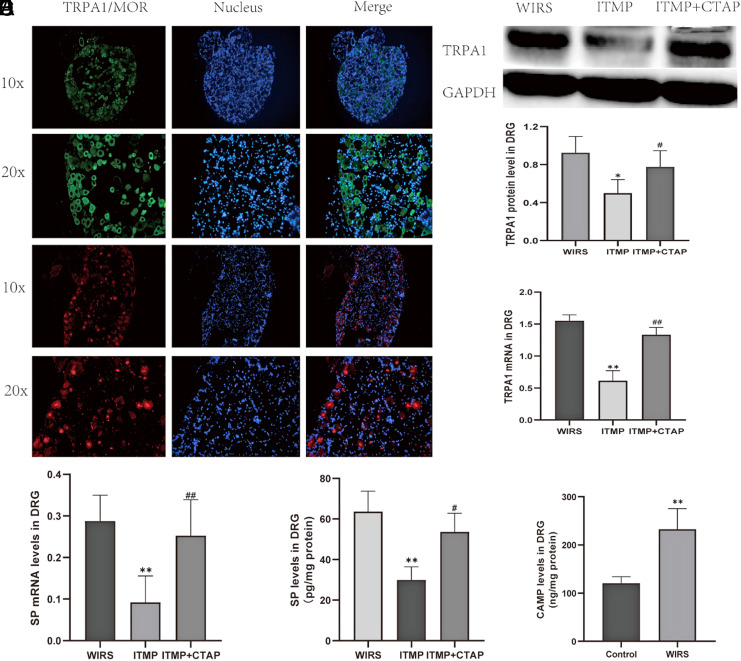
ITMP downregulated the expression of TRPA1 and SP in the DRG, but the regulation could be reversed when combined with CTAP. (A) Distribution of TRPA1 (green) and MOR (red) in thoracic gastric reflected the DRG as demonstrated by immunofluorescence; the nuclei were stained with bright blue. (B) Relative quantification of TRPA1 in the DRG as measured by Western blot. (C) TRPA1 mRNA levels in the DRG as measured by real-time-PCR. (D) SP mRNA levels in the DRG as measured by real-time-PCR. (E) SP levels in the DRG as measured by ELISA. (F) Cyclic adenosine monophosphate levels in the DRG as measured by ELISA. All data are presented as mean ± SEM, n = 6 rats per group; statistical analysis was performed using a Student’s *t*-test, **P < .01, *P < .05 compared to the WIRS group; ##*P*< .01, #*P*< .05 compared to the ITMP group. DRG, dorsal root ganglia; ITMP, intrathecal morphine preconditioning; PCR, polymerase chain reaction; SP, substance P; TRPA1, transient receptor potential ankyrin; WIRS, water-immersion restraint stress.

The results demonstrated that ITMP led to a significant reduction in both TRPA1 mRNA (*P* = 0.0009, Figure 5C) and protein expression (*P* = .0086, Figure 5B); however, this reduction was partially reversed when CTAP was administered intrathecally 10 minutes before ITMP, as indicated by the data presented in (*P* = .003, Figure 5B and *P* = .048, Figure 5C). An experiment using immunofluorescence was conducted to explore the possible co-expression of TRPA1 and µ-opioid receptors in the DRG. The findings revealed that there was indeed co-localization of TRPA1 and µ-opioid receptors in the DRG neurons following exposure to WIRS, as depicted in (Figure 5A). The level of cAMP in the DRG was found to have increased after WIRS from 120.7 ± 13.51 to 232.7 ± 42.7 (*P* = .0024, Figure 5F), and intrathecal administration of H89 was found to reduce WIRS-induced gastric mucosal lesions from 52.7 ± 8.19 to 32.9 ± 5.02 (*P* < .0001, Figure 1D). The data showed that ITMP definitely reduced the levels of SP in the DRG according to both real-time-PCR (*P* = .019, Figure 5D) and ELISA (*P* = .0013, Figure 5E).

## Discussion

In this study, it was found that the regulation of TRPA1/SP contributes to the WIRS-induced AMGL, both in the DRG and gastric mucosa, and this process occurs through a cAMP-dependent signaling pathway. Specifically, both mRNA and protein levels of TRPA1 and SP showed a substantial increase in the DRG and gastric mucosa after exposure to WIRS. The antagonist of TRPA1, HC-030031, was found to significantly relieve gastric mucosal lesions induced by WIRS. Additionally, intrathecal administration of H-89, a PKA inhibitor, was found to significantly relieve gastric mucosa injury induced by WIRS. Furthermore, the data suggested that ITMP suppresses WIRS-induced gastric mucosa injury by inhibiting the activity of TRPA1 in the DRG through a cAMP-dependent signaling pathway. The protective effect of ITMP could be blocked when combined with CTAP, a specific µ-opioid inhibitor. These findings suggest that there is a functional interaction between the µ-opioid receptor and TRPA1.

Accumulating evidence supports that TRP channels significantly contribute to the perception and sensation of pain in the gastrointestinal tract. Among TRP channels, TRPA1 is a non-selective cation channel predominantly found in sensory neurons and nerve fibers. The present study confirmed that TRPA1 is involved in the formation and persistence of WIRS-induced gastric lesions by elevating SP release in both the stomach and DRG neurons, as previously demonstrated by the study investigators.^26^ The presence of TRPA1 in the DRG and gastric mucosa was upregulated significantly, as well as the expression of SP. To further corroborate these aspects, additional investigations were conducted to explore the function of TRPA1 in gastric protection. WIRS upregulated the levels of TRPA1 both in the DRG and gastric mucosa, while HC-030031, as a selective antagonist to TRPA1, showed a definite protective effect against AMGL induced by WIRS, indicating the function of TRPA1 as a target for AGML medication. The conversion of cold sensation to pain, the adjustment of gastric acid secretion and mucosal blood flow were speculated to be involved in the physiological mechanism.

The cAMP signaling pathway serves as a downstream pathway for both TRPA1 and μ-opioid receptors. Based on this relationship, it was hypothesized that the cAMP signaling pathway might be involved in the molecular processes associated with WIRS. Subsequently, experimental findings demonstrated an elevation in cAMP levels within the DRG following exposure to WIRS. In order to investigate the role of the cAMP signaling pathway in WIRS, the impact of intrathecal H-89, a specific PKA inhibitor, was also assessed through administration. An intrathecal administration of H-89 was observed to reduce gastric mucosal lesions induced by WIRS. Prior studies in this field have demonstrated that opioid agonists can induce gastroprotective when administered before WIRS.^23^ In the present study, it was observed that ITMP might exert its gastroprotective effects by potentially suppressing the upregulation of TRPA1 protein expression and mRNA levels in both the DRG and gastric mucosa induced by WIRS. Meanwhile, there was a decrease in the release of SP in the DRG and gastric mucosa, showing an antinociceptive effect against WIRS. In addition, the gastric protection and reduction of TRPA1 expression by ITMP were observed to be reversible with intrathecal CTAP administration, a specific µ-opioid receptor antagonist. The results indicated the presence of µ-opioid receptors and TRPA1 in the gastric-related DRG of rats following WIRS, which indicated that there was an interaction function between MOR and TRPA1. Transient receptor potential ankyrin 1 is recognized as a key factor in initiating and propagating the immune response to stress and has been found to be highly co-expressed with TRPV1 stomach-labeled DRG neurons.^27,28^ Both TRPV1 and TRPA1 are highly co-expressed in different regions of the central nervous system.^11,12^ These polymodal nociceptors play a significant role in various sensory functions, including temperature, mechanical, and chemical sensing, as well as in processes such as neurogenic inflammation and hyperalgesia.^13-15^ Furthermore, these receptors are suggested to potentially exhibit functional interactions with each other in a cAMP/PKA dependent manner.^16,17^ Thus, the current study investigators assumed that there may be some possible functional interactions between TRPA1 and MOR.

This study demonstrated that enhanced cAMP levels in the DRG after WIRS and the administration of the PKA inhibitor (H-89) via intrathecal route demonstrated a gastroprotective effect, suggesting the potential involvement of a cAMP/PKA-dependent pathway in the DRG in modulating the development of gastric injury induced by WIRS. These findings highlight the significance of cAMP/PKA signaling within the DRG as a key molecular mechanism contributing to the development of gastric injury under WIRS conditions.

Our study has limitations. First, while animal models are valuable for preliminary investigations, findings may not always directly translate to human responses. Second, WIRS is a single stressor model used in this study. In real-life scenarios, individuals may experience a combination of stressors, and the effects of morphine on TRPA1 and related pathways may differ in multifactorial stress conditions. Third, investigators speculated that the cAMP/PKA pathway might be involved in the manner by which morphine modulates the expression of TRPA1 induced by WIRS. However, additional investigations are needed to thoroughly explore and substantiate this possible association in future studies. Additional investigations will be essential to gain deeper insights into the precise mechanisms and interactions underlying the modulation of TRPA1 expression by morphine and its association with the cAMP/PKA pathway in the context of WIRS.

These study findings indicate that the suppression of the increase of TRPA1 in the DRG is crucial for the effectiveness of ITMP in providing gastric protection. This highlights the importance of TRPA1 as a potential target for understanding and potentially mitigating the gastric damage associated with WIRS. Further research in this area could provide valuable insights into the underlying mechanisms and may pave the way for the development of innovative therapeutic approaches for AMGL and related disorders.

## Figures and Tables

**Table 1.** Histopathological Gastric Mucosal Lesions Score

	Description	Score
Ulceration	Without	0
	Superficial	1
	Giant ulcer	2
Damage in mucous layer	Without	0
	Local epithelial cell necrosis and abscission	1
	Epithelial cell necrosis and abscission	2
	Glandular atrophy and reduction	3
Hyperemia/edema	Without	0
	Mild	1
	Moderate	2
	Severe	3
